# Cryogenic Gas-phase IR Spectroscopy on a Commercial Ion Mobility-Mass Spectrometry Platform

**DOI:** 10.1021/acs.analchem.5c08001

**Published:** 2026-05-08

**Authors:** Gergo Peter Szekeres, Jacob S. Jordan, Jerome Riedel, Jan Horlebein, Gurpur Rakesh D. Prabhu, Michael Götze, Steven Daly, Stephan Warnke, Kevin Pagel

**Affiliations:** † Department of Chemistry, Biochemistry, and Pharmacy, 9166 Freie Universität Berlin, Altensteinstraße 23A, Berlin 14195, Germany; ‡ 28259 Fritz-Haber-Institut der Max-Planck-Gesellschaft, Department of Molecular Physics, Faradayweg 4-6, Berlin 14195, Germany; § MS Vision, Televisieweg 40, Almere 1322 AM, The Netherlands; ∥ Isospec Analytics SA, Rue de Lausanne 64, Renens 1020, Switzerland

## Abstract

The combination of gas-phase infrared (IR) spectroscopy with ion mobility spectrometry and mass spectrometry provides detailed, multidimensional structural information that facilitates the identification of unknown analytes. The instrumentation for performing these measurements is typically home-built and requires substantial expertise to operate. Here, we demonstrate messenger-tagging IR spectroscopy on a modified Synapt G2-S ion mobility-mass spectrometer (IM-MS). Messenger-tagging is performed in a commercially available cryogenic ion trap inserted between the exit of the transfer cell and the TOF pusher assembly, and tagged ions are excited by IR light before MS measurement. We report the adjustments to the timing cycle and voltage gradient in the Synapt G2-S necessary for efficient trapping and messenger-tagging in the cryogenic trap. The capabilities of this instrument are demonstrated by measuring IM-MS-IR data for leucine enkephalin, a benchmark standard in mass spectrometry. The ability to selectively transmit ions of specific mobility enables the separation and subsequent IR spectroscopy of the isomeric trisaccharides cellotriose and melezitose. These data demonstrate the first implementation of messenger-tagging IR spectroscopy in a widely used, commercially available IM-MS system. The user-friendly implementation of these techniques overcomes a significant barrier to the widespread incorporation of orthogonal IR spectroscopy measurements in existing IM-MS workflows and will aid in distinguishing unknown molecules in untargeted -omics measurements.

## Graphical abstract

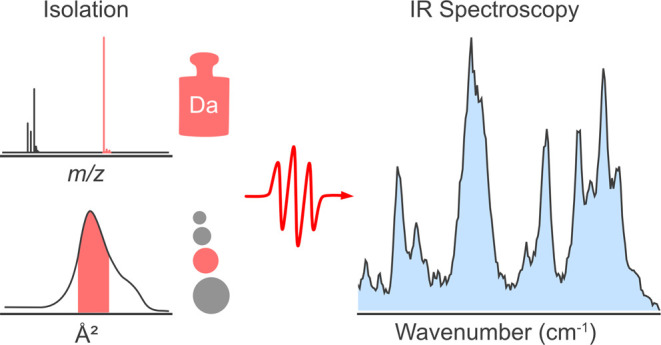

## Introduction

The development of multidimensional separation methods coupled to mass spectrometry has dramatically increased the throughput and specificity of omics workflows. Hyphenated systems involving liquid- and gas-phase separation followed by tandem-MS analyses enable access to detailed structural information. Ion mobility spectrometry, which separates ions on the basis of their collision cross-section in the gas phase, has proven particularly useful for the determination of biomolecular structure and the separation of isobaric molecules on very short time scales.^1–4^ Modern instruments combining ion mobility and mass spectrometry (IM-MS) approach the resolution of traditional liquid chromatography separation but on orders of magnitude shorter time scales (<1 s as opposed to minutes).^5^ These instrumental advancements have spread to enable higher-throughput analyses in the fields of metabolomics, lipidomics, and proteomics.^6–8^

IM-MS is increasingly applied in the field of glycomics,^9^ where the characterization of glycan isomers presents a significant analytical challenge. In several cases, IM-MS has enabled the differentiation of glycan isomers based on differences in their collision cross-section alone on time scales much faster than corresponding condensed-phase techniques.^5,10,11^ However, isomeric glycans can often have collision cross-sections with a difference close to the resolution limit of the instrument, preventing the unambiguous structural assignment of each analyte. While analyte-specific calibration methods can improve the certainty of structural assignments in such cases,^12^ these are labor-intensive and only suitable for focused investigations. Thus, for higher-throughput analyses of unknown mixtures, an additional analytical dimension providing structure-specific information is essential.

Gas-phase vibrational spectroscopy yields such structure-specific information in the form of a vibrational fingerprint for each molecule.^13,14^ While conventional scattering- or transmission-based methods typically employed in condensed-phase experiments are rarely suited for the low analyte density in the gas phase, several methods approach this problem from a different perspective: by looking at the effect of the light on the ion rather than the effect of the ion on the light. These approaches are collectively termed action spectroscopy. Vibrational action spectroscopy is most commonly performed by monitoring the fragmentation of a molecule after the consecutive absorption of multiple IR photons.^15–17^ This technique, called IR multiple-photon absorption dissociation (IRMPD) or IR ion spectroscopy (IRIS), requires high pulse energies, which has historically limited experiments in the fingerprint region (<1800 cm^–1^) to sophisticated light source infrastructures, such as free-electron lasers. Since the 1990s, benchtop laser systems sufficient for IRMPD have become increasingly available,^14,18^ which has enabled IRMPD action spectroscopy to be coupled to many different home-built and commercial mass spectrometry systems.^19–21^ However, it was not until recently that the combination of IRMPD spectroscopy with a commercial ion mobility-mass spectrometer was demonstrated for the first time.^18^

In the gas phase, the absorption of multiple photons results in an increase in the internal temperature of the ion before fragmentation. This can increase spectral complexity by causing peak shifts and the broadening of vibrational modes. To mitigate these, it is necessary to maintain ions at low internal temperatures during the measurement. One possible approach is to embed them in an inert cooling matrix, such as superfluid helium nanodroplets.^22,23^ When the embedded ions absorb photons, energy is dissipated by droplet evaporation. Spectra are produced by monitoring the appearance of bare ion signal as a function of laser wavelength. This technique delivers exceptionally narrow peaks, but requires substantial expertise to operate, and the necessary peak powers and laser pulse structure generally limit the availability of this technique to free electron laser facilities.

An alternative cryogenic approach is known as messenger-tagging IR spectroscopy or cryogenic IR ion spectroscopy (CIRIS). Ions are cooled to cryogenic temperatures (usually <50 K) while stored in an ion trap, and an inert gas (typically N_2_ or H_2_)^24–26^ is released into the trap at low partial pressure as a tag molecule. At temperatures where the thermal energy is significantly lower than the ion-tag binding energy, weakly bound complexes between the ion and the tag can be formed. The absorption of a single photon via vibrational excitation is generally sufficient to break this interaction, resulting in the dissociation of the messenger tag, and this can be detected by a depletion of the tagged ion complex. This approach requires lower pulse energies compared to IRMPD and can be readily implemented with benchtop laser systems covering the majority of the spectral range of interest.^26,27^ These benefits have led to an expanding application of CIRIS both in fundamental physical chemistry experiments and in application-oriented research.^14,26,28,29^

The routine use of messenger-tagging IR spectroscopy in analytical workflows has been limited due to the need for specialized, home-built instrumentation and highly trained operators. Prior coupling of structures for lossless ion manipulation (SLIM) ion mobility spectrometry (IMS) approaches to conventional Agilent QTOF instrumentation has shown success for the acquisition of peptide IR spectra in a more user-friendly format.^30^ To expand this technique to a broader range of mass spectrometers, we demonstrate the modification of a commercial Synapt G2-S IM-MS platform for messenger-tagging IR spectroscopy by incorporating a cryogenic ion trap after the ion mobility region. The choice to modify a Synapt G2 is based on the broad use of this instrument in the MS field, the wide availability of this instrument on the second-hand market, the encyclopedia of resources available for teaching instrument operation, and previous examples of successful modification of this instrument platform for spectroscopy measurements.^31–33^ The resulting instrument, named the Cold Photo Synapt, enables mobility separation of analytes prior to IR spectroscopy and mass measurement in a commercial instrument platform familiar to mass spectrometrists and general analytical scientists.

### Modifications to the Synapt G2-S

The instrument is based on a commercial Synapt G2-S mass spectrometer modified with a cryogenic ion trap (Isospec Analytics SA, Renens, Switzerland).^14,34^ This modified instrument has the capacity for quadrupole mass selection of ions based on user-defined *m*/*z* values, the collection of traveling wave IM data, slicing of specific arrival time windows in the mobilogram for analyte separation based on drift time, storage and N_2_-tagging of ions in the cryogenic ion trap, irradiation by an IR laser, and mass measurement in a single instrument (Figure 1). Readers may find the schematic for the unmodified version of the instrument helpful for the following discussion (Figure S1).

**Figure 1:**
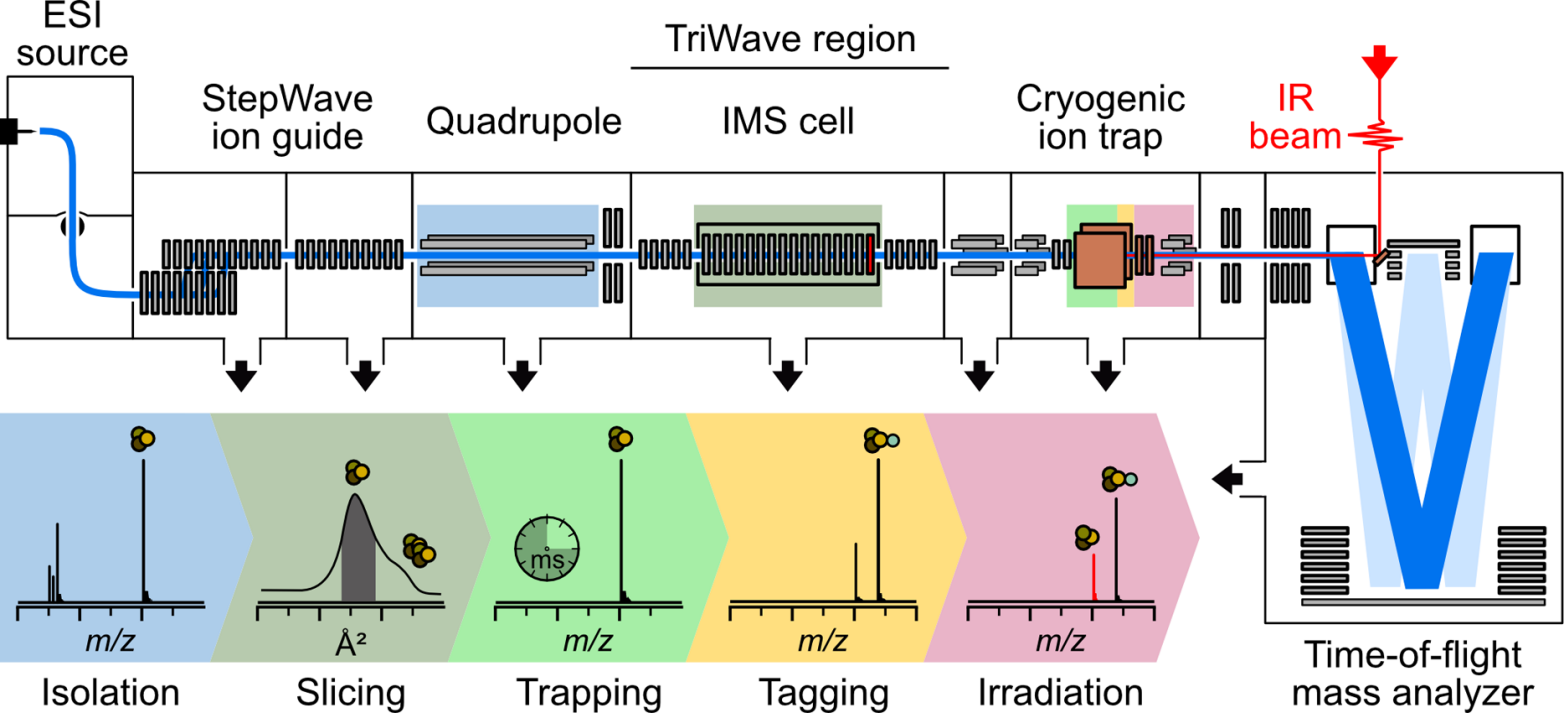
A schematic representation of the instrument design detailing the modifications and measurement capabilities of the instrument. A commercially available cryogenic ion trap was incorporated between the end of the TriWave region and the TOF pusher assembly. Optical access to the cryogenic trap is provided by a KBr window positioned in the TOF lid above the pusher assembly.

### Ion Mobility Slicing

The region upstream of the ion mobility cell is unmodified. Specific arrival time windows in the ion mobilogram can be isolated by varying the voltage on the exit lens of the mobility cell in the TriWave region (Figure 1, bright red lens in the IMS region). For transmission of all species in the mobilogram, the voltage is fixed to a given value to produce a downhill gradient relative to the preceding ion optics in this region. For IMS slicing, the voltage of the exit lens is increased by an absolute value of +50 V (in positive mode) to prevent transmission of unwanted species and returned to the normal value for transmission at a user-specified arrival time and duration. All ions that pass the exit lens are transmitted through the unmodified transfer cell into the cryogenic ion trap for messenger-tagging and IR spectroscopy. All instrument parameters upstream and downstream of the modified region are controlled in MassLynx V4.1 (Waters Corporation, Milford, MA).

### Cryogenic Ion Trapping and Infrared Spectroscopy

The cryogenic ion trap was placed between the exit of the transfer cell and the entrance of the pusher region by MS Vision (Almere, NL) (Figure 1). The cryogenic ion trap is connected to a closed-cycle helium cryostat (single-stage 0/40 coldhead, Oxford Cryosystems, Long Hanborough, United Kingdom), maintained between 30 and 45 K (adjusted for optimal tagging of different analytes) using a temperature controller (Model 336, Lake Shore Cryotronics, Westerville, OH, USA). The heat transfer to the trap is established through conductive copper plates positioned between the trap and the cold head.

The cryogenic trap is described in detail elsewhere.^14^ In brief, it consists of a pair of printed circuit boards containing 62 electrodes, which are individually connected for complete control over the DC potential within the trap (Figures 1 and S2). Einzel lens stacks are positioned at the entrance and exit of the ion trap for ion focusing. Two RF voltages (180° shifted in phase with respect to each other) are supplied to confine ions within the trap (MIPS Ultra Power High Q Head, GAA Custom Electronics LLC., Kennewick, WA, USA). Additional DC electrodes on the side of the ion trap ensure the lateral confinement of ions. The voltage difference between the side and pad electrodes within the trap can be modulated to excite or cool ions, thereby modulating the ion tagging efficiency on the fly. DC voltages for all ion trap optics are supplied by two Modular Intelligent Power Supplies (MIPS) (GAA Custom Electronics, LLC., Kennewick, WA, USA), set via a custom GUI, and applied to ion trap elements via a custom circuit board and connectors. The ions are transferred into and out of the trap by three hexapole ion guides installed during instrument modification, two before and one after the ion trap. Three additional turbomolecular pumps (two Edwards EXT255H (220 L s^–1^) and one Edwards nEXT300 (300 L s^–1^), Edwards Ltd., Burgess Hill, United Kingdom) were also added to this region for three main purposes: (i) to add a differential pumping stage between the exit of the IMS region and the cryogenic ion trap (ii) to ensure that the tagging gas can be efficiently pumped away after the allowed tagging time during each trap cycle, and (iii) to ensure that low pressure is maintained in the TOF region of the instrument. During normal operation without trapping, the pressure in this region is maintained at ∼1 × 10^–7^ mbar (with no trapping gas pulsed in) and a linear DC gradient is maintained throughout the trap to promote transmission of ions to the pusher as a coherent bunch.

A trapping cycle consists of three phases: ion accumulation and tagging, irradiation, and release from the cryogenic trap to the TOF analyzer (Figure S3). In normal operation with a 10 Hz pulsed laser system (detailed below), this cycle is repeated twice per laser pulse (i.e., at 20 Hz): one with and one without laser irradiation. Acquiring spectra without irradiation accounts for changes in both the overall signal intensity and the tagging efficiency over the course of an experiment. As the ion mobility cycle time is affected by the user-defined *m*/*z* range, the option “Enable mobility separation delay after Trap Release” was selected in MassLynx and the delay was adjusted to obtain an ion mobility cycle of exactly 10 or 25 ms for compatibility with a 20 Hz trap cycle. Thus, 2–5 ion mobility cycles were accumulated per trapping cycle, and a complete IR acquisition cycle at each wavelength, including two trapping cycles, a laser pulse, and the acquisition of two triggered TOF traces, was set to 100 ms.

A trapping cycle is initiated using the IMS start trigger from the instrument as a start signal. This signal is passed to two MIPS devices which control the timing of the trap optics. During the first accumulation period (0–50 ms), the exit lens of the cryogenic ion trap is initially maintained at +25 V (in positive mode) and a mixture of 20% N_2_/80% He is pulsed into the chamber via a solenoid valve (Parker Hannifin GmbH, Kaarst, Germany) 2 ms prior to ions entering the ion trap. The combination of a high uphill potential and the gas in the ion trap thermalizing ions enables them to be stopped after ejection from the transfer cell, and facilitates their confinement. The average pressure reading inside the trap chamber during typical operation is ∼2 × 10^–6^ mbar but can be adjusted to higher or lower values by changing the gas pulse duration to aid in thermalizing ions with different masses. As ions are thermalized to cryogenic temperatures, N_2_-tagged species with an *m*/*z* shift equivalent to +28 Da from the molecular ion are formed (Figure 2A). For 50 ms, ions are accumulated in the cryogenic ion trap, thermalized, and tagged after which they are released by lowering the voltage of the final lens element in the trap from +25 V to −50 V (in positive ion mode), accelerating the ions through the exit Einzel lens stack, the third hexapole ion guide, and into the pusher assembly.

**Figure 2:**
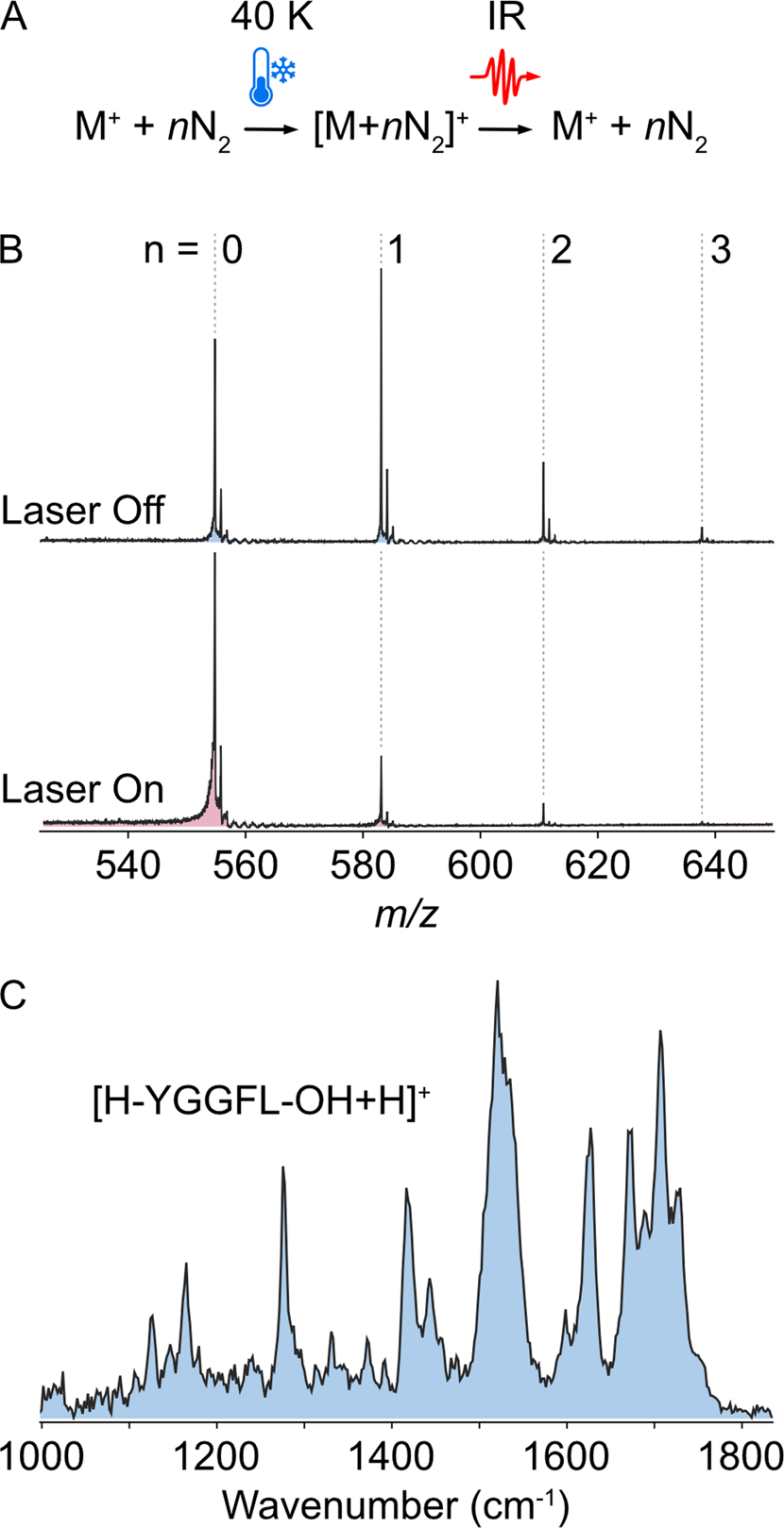
Messenger-tagging IR spectrum of protonated leucine enkephalin monomer ions acquired using the Cold Photo Synapt. (A) Scheme showing tagging and detagging of ions; (B) mass spectra of trapped and tagged protonated leucine enkephalin monomer ions with the laser off (blue) and laser on (red) at 1688 cm^–1^ where a high extent of depletion is observed. The numbers above *m*/*z* peaks indicate the number of N_2_ tags on each ion. (C) Messenger-tagging IR spectrum of the protonated leucine enkephalin monomer.

In the unmodified Synapt G2-S instrument, the pusher frequency is controlled by the IMS start trigger and calibrated for each instrument to include the measured flight time of ions from the end of the IMS cell to the pusher. However, the trapping of ions in the cryogenic ion trap after IMS interferes with this timing scheme. As a result, it was necessary to manually define the timing of the TOF pusher to match the time of ions reaching the pusher after ejection from the cryogenic ion trap. This was performed by enabling the “Targeted Enhancement Mode” function in MassLynx. In this mode, the pusher was continuously triggered at a frequency of 18 kHz between 45 and 99 μs (50.045 to 50.099 ms in the trap cycle) after ion release from the cryogenic trap by a trigger signal sent from the MIPS devices. This timing must be adjusted to account for the different times-of-flight for ions with different *m*/*z* values because time-of-flight is proportional to the square root of analyte *m*/*z*. However, these values are constant under constant voltage gradient conditions and thus a calibration curve from *m*/*z* to time-of-flight can be constructed for automated adjustment of the pusher timing. Here, the optimal timing was determined by measuring the abundance of the ion of interest as a function of the delay between the ion release from the cryogenic ion trap and triggering of the pusher. During this time, ions can already begin to accumulate in the cryogenic ion trap for the next trap cycle.

The IR pulses (see details below) enter the instrument on top of the TOF region^31–33^ through a KBr optical window. The beam is reflected by a 45° gold-coated flat mirror embedded within the pusher assembly that aligns the laser beam collinear with the ion trajectory. At 50.092 ms, into the trap cycle, the IR laser irradiates the ion packet. If the ion has a transition that is resonant with the IR photons at a given wavelength, the energy gain due to photon absorption is redistributed within the ion, and the N_2_ tag dissociates from the ion, resulting in an increase in the signal intensity of untagged species (Figure 2A). Irradiation of ions after release from the trap reduces nitrogen retagging, increasing the signal-to-noise ratio in the messenger-tagging IR spectra. Because ions are irradiated during their flight between the trap and TOF analyzer, the timing of laser pulses must be optimized for ions of different *m*/*z*, i.e., their corresponding transit times between the cryogenic trap and the pusher.

The IR acquisition cycle (a single trapping cycle from 0–100 ms, including acquisition of two respective mass spectra with and without laser excitation (Figure 1)) is repeated a user-defined number of times at each laser wavelength to average spectra. This spectral averaging accounts for shot-to-shot differences in laser power and differences in ion signal due to electrospray fluctuations, allowing data to be obtained with sufficiently high signal-to-noise ratios to detect subtle shifts in tagging yield. In a typical experiment, the laser is stepped in 2 cm^–1^ increments between 990 and 1810 cm^–1^ while averaging 75 spectra at each wavelength. In total, this results in an acquisition time of ∼1 h for high resolution, high signal-to-noise spectra. It is important to note that not all analytes will need such high resolution scanning over such a broad range of wavelengths. Experiments can be performed with much higher through-put by limiting the wavelength range to be investigated or by lowering the resolution of the spectral data by increasing the laser wavelength step size.

### Data Acquisition

To acquire individual pusher–puller cycles, we have connected the second output of the multichannel plate preamplifier to a TeleDyne SP ADQ32 digitizer (Thousand Oaks, CA, USA) interfaced with a custom acquisition software written in Python. This software enables spectra from individual pusher–puller cycles to be averaged over multiple trap cycles before saving the averaged data in a memory-efficient binary file format. This software also enables the remote control of the laser, allowing a preset number of scans to be acquired at each laser wavelength, tuned discretely in user-defined wavenumber steps. Importantly, this acquisition interface allows for maximum flexibility in tunable parameters, while still retaining the ability to record IMS and MS data through MassLynx. The non-normalized IR spectra can be displayed in real time or the complete processing of IR data can be performed post-acquisition using a custom Python analysis code.

### IR Spectrum Retrieval from TOF Signal

As the field of messenger-tagging IR spectroscopy is rapidly expanding, it is important to establish a unified consensus for IR spectrum retrieval. Here, we propose an approach that considers fluctuations in ion signal, instrument conditions, and optics.

Messenger-tagging IR spectra were calculated from the baseline-corrected TOF signal of the untagged ion (integrated between user-defined *m*/*z* values) recorded as a function of wavelength (λ). For each wavenumber step, the tag depletion signal (*N*_dep_(λ)), was determined by subtracting the signal intensity without laser irradiation (*I*_OFF_(λ)) from the intensity observed with irradiation (*I*_ON_(λ)): *N*_dep_(λ) = *I*_ON_(λ) -*I*_OFF_(λ). This depletion signal was then normalized to the reference signal, which here corresponds to the laser-off signal of the untagged ion, *I*_OFF_. This normalization yields the depleted fraction: *F*_dep_(λ) = *N*_dep_(λ)/*I*_OFF_.

To ensure a large dynamic range and high signal-to-noise ratio, the laser pulse energies were set to reach a maximum of 90–95% tag depletion (*S*_rel_). This approach avoids direct saturation of tag depletion, but due to probabilistic considerations of saturation (i.e., if *i* out of *N* tagged ions have absorbed a photon and undergone tag depletion, a total of (*N* – *i*) tagged ions remain that can absorb photons in an observable manner in the same cycle) the depleted fraction must be linearized as *S*_rel_(λ) = -*ln*(1-*F*_dep_(λ)). This linearized signal, *S*_rel_(λ), represents the absorption intensity, which must be normalized by the photon flux experienced by the ions to yield the final relative absorption cross-section (σ_rel_(λ)).

The laser energy profile, *E*(λ), was recorded using a pyroelectric power sensor (Ophir Vega, Ophir Optronics, West North Logan, UT, USA), and converted to a photon flux profile. The number of photons at a given wavelength is proportional to the measured average pulse energy divided by the photon energy (*E*_photon_ = hc/λ). Considering a near-diffraction-limited laser focus (i.e., *A*∝λ^2^) that overlaps with the ion cloud, the photon flux is proportional to (*E*(λ)·λ)/λ^2^ = *E*(λ)/λ. The final relative absorption cross-section, σ_rel_(λ), was obtained by normalizing the linearized intensity to this photon–flux profile, according to

$$\sigma_{rel}=\frac{-\lambda \cdot \ln\left(1-\frac{I_{ON}\left(\lambda \right)-I_{OFF}\left(\lambda \right)}{S_{rel}}\right)}{E\left(\lambda \right)}$$

where *S*_rel_ was chosen as the integrated untagged ion signal in the same acquisition cycle without laser excitation, i.e., *I*_OFF_(λ).

## Methods

### Sample Preparation

The peptide leucine enkephalin was dissolved to a final concentration of 20 μM in a 1:1 mixture of water/methanol. Melezitose and cellotriose mixtures were prepared at 10 μM of each sugar in 1:1 water/methanol with 400 μM NaCl to ensure adduct formation. All chemicals were purchased from Sigma-Aldrich (St. Louis, MO, USA) and used without further purification.

Ions are formed by nanoelectrospray from ∼1.2 μm borosilicate emitters^35,36^ pulled in-house using a Sutter Instrument P-1000 micropipette puller (Novato, CA). An electrospray capillary voltage of ∼0.7–1.2 kV was applied to solutions via a platinum wire inserted into the emitter and in contact with the solution.

### Tunable Mid-IR Light Source

The IR laser used in these experiments is a LaserVision optical parameteric oscillator/optical parametric amplifier (OPO/OPA) system with an additional zinc germanium phosphide crystal-based difference frequency generation module that generates tunable mid-IR pulses (LaserVision, Bellevue, WA). The system is pumped by a 1064 nm Nd/YAG laser (Surelite EX) at a repetition rate of 10 Hz, and can be tuned across the entire mid-IR spectral range of interest with pulse energies of 1.5 mJ and 3.5 mJ at 10 μm and 5 μm, respectively. The laser pulses were synchronized to the trapping cycle via a delay generator (DG645, Stanford Research Systems, Sunnyvale, CA, USA), which was also used to trigger and set the Q-switch delay of the pump laser.

## Results and Discussion

### Acquisition of IMS, IR, and MS Data from a Single Modified Commercial Instrument

This instrument enables the acquisition of messenger-tagging IR spectra in tandem with ion mobility-mass spectrometry measurements in a user-friendly format familiar to most analytical chemists. To demonstrate the utility of this new instrument, we first measured the peptide leucine enkephalin, a commonly used biomolecular standard for mass spectrometry.

Tagging mass spectra of 20 μM leucine enkephalin with the laser off and on in positive ion mode are shown in Figure 2B. At 40 K and a gas pressure of ∼2 × 10^–6^ mbar in the ion trap (optimized before each measurement for best conditions), leucine enkephalin has ∼1 N_2_ tag with a tagging yield >50% on average, which provides sufficient dynamic range for observing tag depletion as a function of excitation wavelength. Increasing the pressure of tagging gas resulted in higher signal intensity, likely due to better thermalization and confinement in the trap. However, this also resulted in lower tagging yields, most likely due to a higher frequency of gas-ion collisions during ejection from the trap resulting in detagging. IMS data from transmitted ions (without trapping) are in agreement with prior reports (Figure S4),^37^ indicating that the addition of the cryogenic ion trap does not induce a measurable change in flight time from the end of the IMS cell to the pusher or result in a significant expansion of the ion packet after IMS separation. While tagging in negative ion mode is often considered challenging due to the messenger tag usually interacting with positively charged moieties, we readily observe the formation of messenger-tagged anions on this instrument (Figure S5). This opens the possibility to acquire IR spectra of negatively charged ions on this modified commercial instrument platform.

Messenger-tagging IR spectra are reconstructed from the ratio of untagged ion abundance in consecutive trapping cycles with and without IR excitation. These measurements were performed on the mass- and mobility-selected protonated leucine enkephalin monomer ions shown in Figure 2B to generate the IR spectrum in Figure 2C. The bands observed in these data compare well with those in spectra previously collected by related gas-phase IR methods.^22,38,39^ These IR spectra showcase the capability of this instrument to simultaneously acquire MS, IMS, and IR spectra in a single setup based on a modified, commercially available instrument platform. This instrument significantly advances the possibility for adaptation of IR spectroscopy as an additional analytical modality in mass spectrometry laboratories.

### Ion Mobility Slicing Enables the Selection of Specific Isomers

Specific arrival time windows in the ion mobilogram can be sliced for subsequent analysis by IR spectroscopy, which enables individual isomers of isobaric ions to be isolated in the gas phase and interrogated separately. To demonstrate this function, a mixture of melezitose and cellotriose, trisaccharide isomers that can be partially resolved by TWIMS,^40^ was investigated (Figure 3A). To enhance IMS separation, the *m*/*z* range was reduced to 400–2500 *m*/*z*, resulting in an IMS cycle of 10 ms after adjustment with the “Mobility delay after trap release” function. On the Synapt G2-S, the number of arrival time bins per IMS cycle is constant, but the overall time per IMS cycle can vary depending on the desired *m*/*z* range input by the user. This change in IMS cycle time (and thus resolution) is performed to account for the slower elution time of heavier analytes from the IMS cell. With a high *m*/*z* limit of 2500 *m*/*z*, the IMS cycle time is reduced to <10 ms, resulting in ∼0.11 ms per bin compared to the ∼0.22 ms per bin obtained with higher *m*/*z* ranges. The higher IMS time resolution obtained under these conditions enables enhanced separation of peaks with similar mobilities.

**Figure 3:**
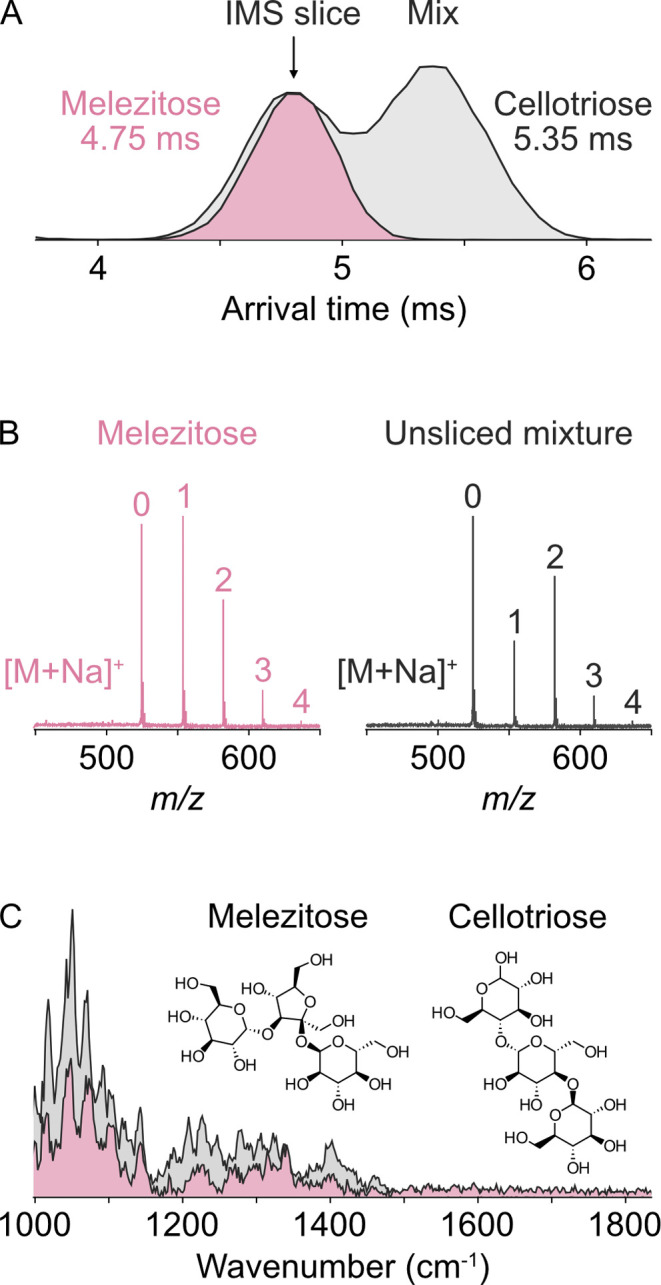
Selection of specific isomers by ion mobility slicing prior to messenger-tagging IR spectroscopy. Representative (A) IMS, (B) MS, and (C) IR spectra obtained from a 1:1 mixture of the trisaccharides melezitose and cellotriose. The numbers in panel B correspond to the number of N_2_ tags on each ion. Red traces correspond to (A) IMS slicing and (C) acquisition of IR spectra on melezitose isolated in the gas phase. The black trace in (C) corresponds to the IR spectrum of the mixture of isomers shown in gray in (A).

A mass spectrum of a 1:1 mixture of melezitose and cellotriose is dominated by the sodium-adducts at *m*/*z* = 526.4 (Figure 3B). Ion mobility data are acquired without the tagging gas mixture in the cryogenic trap (i.e., at 1 × 10^–7^ mbar) to maintain a compact ion packet after release from the mobility region and to determine the time and duration over which to trigger IMS slicing. IMS data must always be acquired without trapping gas in the trap region in order to determine the drift time at which to slice unknown ions. The mobilogram for this *m*/*z* contains two partially resolved peaks at arrival times of ∼4.75 ms and ∼5.35 ms (Figure 3A, black trace). Based on prior reports from the same mixture of isomers on a Synapt G2-S instrument,^40^ we assign the earlier arrival time peak as melezitose and the latter as cellotriose. The FWHM of these peaks is ∼0.4 ms, similar to that obtained in prior experiments by Hoffman et al., where peak widths of ∼0.5 ms (∼3 bins) were obtained.^40^ Importantly, these data indicate that the inclusion of the cryogenic ion trap does not result in a significant expansion of the ion packet between the end of the ion mobility cell and the TOF pusher, which would result in an increase in the peak width. IMS slicing of a 0.5 ms window centered on 4.75 ms results in the effective isolation of the melezitose isomer from the mixture in the gas phase (Figure 3A, red trace).

Following mobility separation and slicing, messenger-tagging IR spectra were acquired. Different isomers can have different extents of tagging, as the messenger-tagging interaction is dependent on localized charge density, which varies depending on the conformation of different isomers. Tagging mass spectra for IMS-sliced melezitose and the unsliced mixture are shown in Figure 3B and have, on average, ∼1 and ∼2 N_2_ tags, indicating different extents of tagging. The different tagging yields observed between isomers may reflect a tagging dependence on the local charge density of the ion or the conformation of specific functional groups, which may be explored in future experiments on this platform to obtain insights into the fundamental mechanisms guiding messenger-tagging at cryogenic temperatures.

A messenger-tagging IR spectrum acquired on IMS-sliced melezitose ions is shown in Figure 3C (red trace). In contrast, the IR spectrum acquired on the unsliced mixture (Figure 3C, black trace) exhibits clear differences in the relative intensities and bands throughout the spectrum. In the region below 1200 cm^–1^, primarily populated by C–O deformations, several bands in the pure melezitose spectrum appear shifted compared to the IR spectrum of the mixture. As visible in Figure 3A, due to differences in ionization and/or transport efficiency between these ions, a somewhat smaller amount of melezitose is present in the gas phase than cellotriose, leading to the apparent band shifts of identical modes in the IR spectrum of the mixture as opposed to simple broadening. These results demonstrate the potential for IM-MS-IR in glycoscience: isobaric species with strong chemical similarities and featuring only C–C, C–H, C–O, and O–H bonds can be separated, isolated, and selectively probed in the gas phase, where minute differences in bond order and corresponding structural differences translate to readily detectable changes in IR spectral features.

The capacity to do these measurements on a commercial instrument modified with a commercially available cryogenic ion trap makes IR spectroscopy amenable to broad adoption, providing a new analytical modality for the identification and characterization of unknown complex molecules, including isomeric glycans and metabolites relevant to disease.

## Conclusions

The combination of MS, IMS, and messenger-tagging IR spectroscopy can provide a wealth of information about the chemical characteristics of hard-to-distinguish analytes such as oligosaccharide isomers. Thus far, existing instruments for performing these measurements together are custom apparatus which boast exceptional acquisition speeds,^41^ but are available only in very few laboratories and generally require a high level of expertise to operate. Here, we have adapted a commercial Synapt G2-S instrument for messenger-tagging IR spectroscopy by incorporating a commercially available cryogenic ion trap. Although the acquisition speed of this device is slower than custom apparatus, the sensitivity, as well as the MS and IMS resolution, are similar to that of custom instruments by nature of being developed on a gold-standard commercial instrument platform. The wide availability of the Synapt G2-S instrument means that many analytical laboratories can modify their instrument to perform cryogenic gas-phase IR spectroscopy, while the well-established operating principles of this instrument and the ability to tune optics voltages in the familiar MassLynx interface will significantly reduce the expertise and training necessary to begin using this instrument for routine analytical purposes.

The current work uses a 100 ms trapping cycle to align with the 10 Hz repetition rate of the mid-IR laser used in these experiments. The trapping cycle could be accelerated to ∼20 ms with similar extents of tagging. Thus, lasers with higher repetition rates can be used for faster acquisition times. With the use of different laser systems with rapid scanning and higher repetition rates (or continuous-wave sources), as well as automated adjustment of instrument parameters and timings to target ions of different *m*/*z* values, it is feasible that gas-phase IR spectrum collection could align with liquid chromatography time scales,^14,42^ aiding in the unambiguous identification of important, complex molecules like glycans or metabolites involved in disease progression. Moreover, given the extended *m*/*z* ranges accessible with the Synapt G2-S, gas-phase IM-MS-IR studies of larger biopolymers, including proteins and nucleic acids, are also possible. Thus, this instrument has the capacity to cover the entire breadth of molecular classes of interest for bioanalytical scientists.

## Supplementary Material



## Notes

A schematic of the unmodified Synapt G2-S; A schematic depicting the DC voltage profile across the cryogenic ion trap; A timing scheme depicting the events during a typical trapping cycle; IMS data of protonated leucine enkephalin ions acquired without trapping; Tagging mass spectra acquired in negative ion mode for deprotonated leucine enkephalin (PDF)
