# Supplementary material for: Cryogenic Gas-phase IR Spectroscopy on a Commercial Ion Mobility-Mass Spectrometry Platform
Source: Anal Chem. 2026 May 8;98(20):14800–8. doi: 10.1021/acs.analchem.5c08001 (PMC13217366; doi:10.1021/acs.analchem.5c08001)
Supplement: Supplementary file 1 [file ac5c08001_si_001.pdf]

Supporting Information
for
Cryogenic Gas-Phase IR Spectroscopy on a Commercial Ion Mobility-Mass Spectrometry Platform

For Submission to: *Analytical Chemistry*

Gergo Peter Szekeres,‡[1,2] Jacob S. Jordan,‡[1,2] Jerome Riedel,[1,2] Jan Horlebein,[2] Gurpur Rakesh D. Prabhu,[1,2] Michael Götze,[1,2] Steven Daly,[3] Stephan Warnke,[4] Kevin Pagel*[1,2]

[1]*Freie Universität Berlin, Department of Chemistry, Biochemistry, and Pharmacy, Altensteinstraße 23A, Berlin, Germany 14195*

[2]*Fritz-Haber-Institut der Max-Planck-Gesellschaft, Department of Molecular Physics, Faradayweg 4-6, Berlin, Germany 14195*

[3]*MS Vision, Televisieweg 40, Almere, The Netherlands 1322 AM*

[4]*Isospec Analytics SA, Rue de Lausanne 64, 1020 Renens, Switzerland*

‡ These authors contributed equally.

*to whom correspondence should be addressed

Email: kevin.pagel@fu-berlin.de

Contents

Figure S1. Schematic of the unmodified Synapt G2-S S3
Figure S2. Schematic of the cryogenic ion trap with DC voltage profile S4
Figure S3. Schematic timing sequence of an IR measurement cycle S5
Figure S4. Ion mobility data for leucine enkephalin acquired without trapping S6
Figure S5. Tagging mass spectra acquired in negative ion mode for deprotonated leucine enkephalin S7

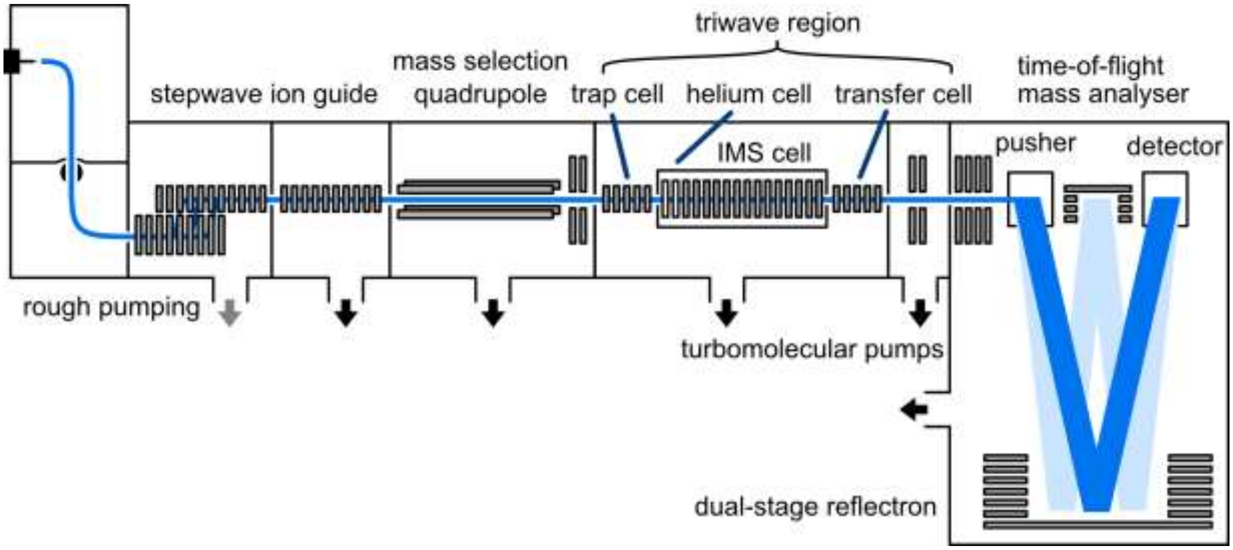

Figure S1. Schematic of the unmodified Synapt G2-S.

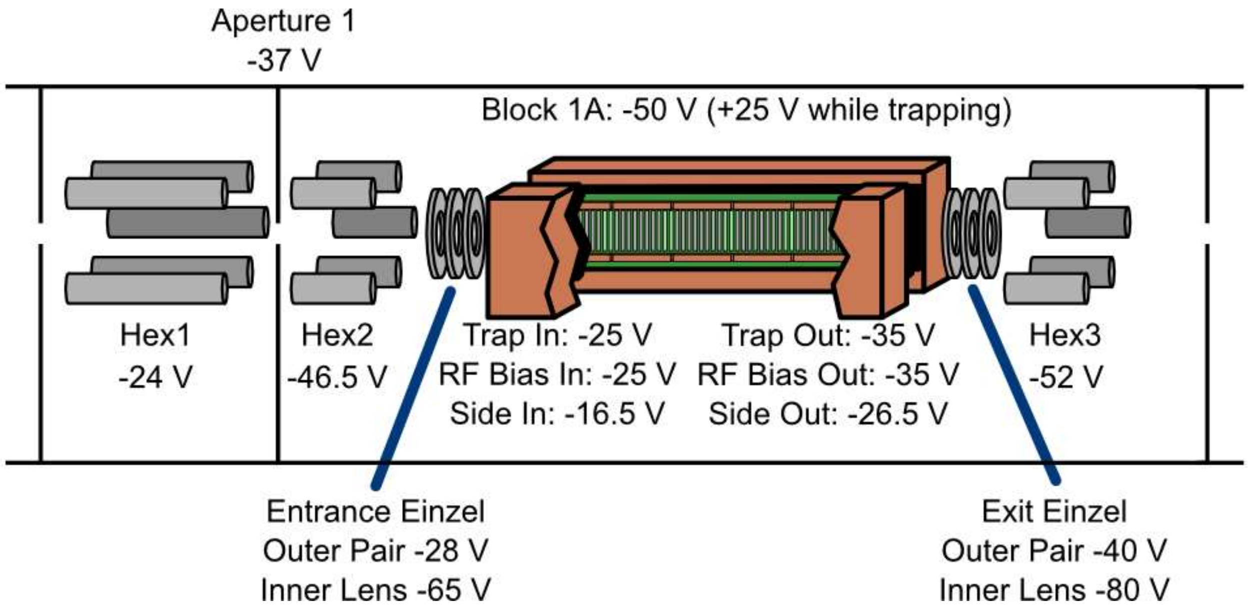

Figure S2. Schematic of the cryogenic ion trap including the DC profile during transmission and trapping.

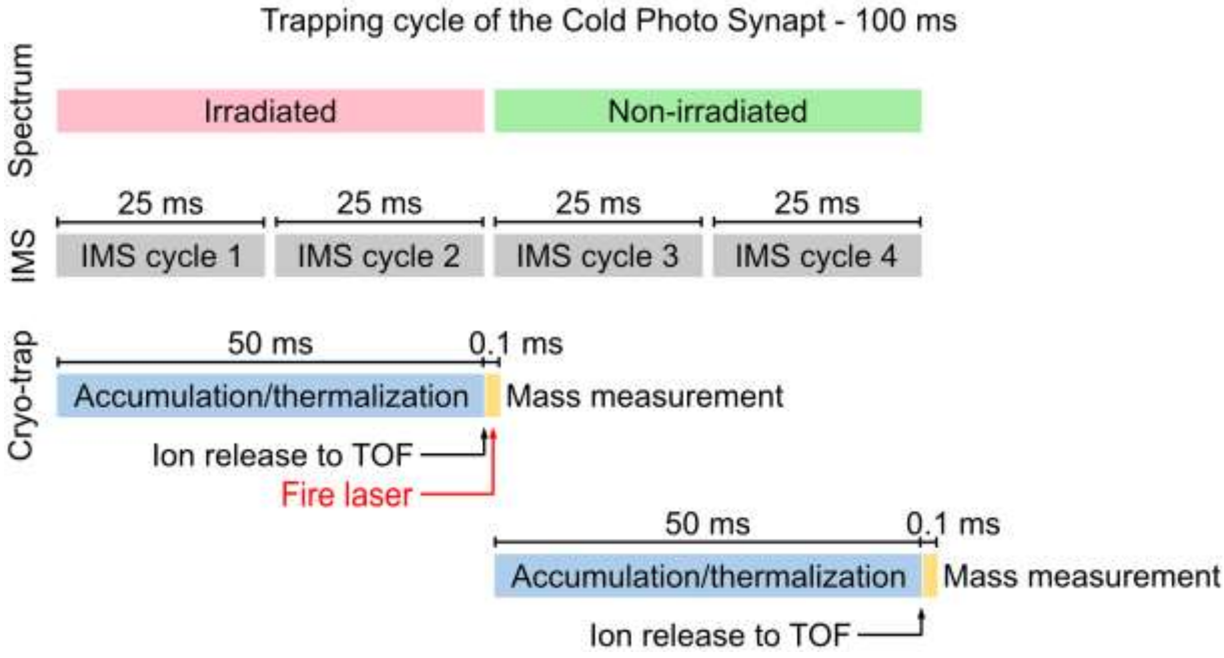

Figure S3. Schematic detailing the sequence of events for the IMS cell and the cryogenic ion trap in a single IR acquisition cycle (~100 ms).

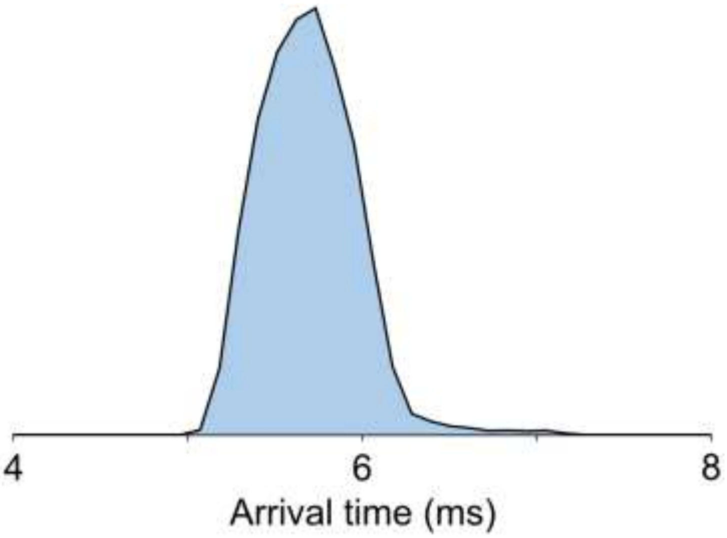

Figure S4. IMS data for MS/MS isolated protonated leucine enkephalin. The drift time and FWHM of the peak is similar to that reported from literature.

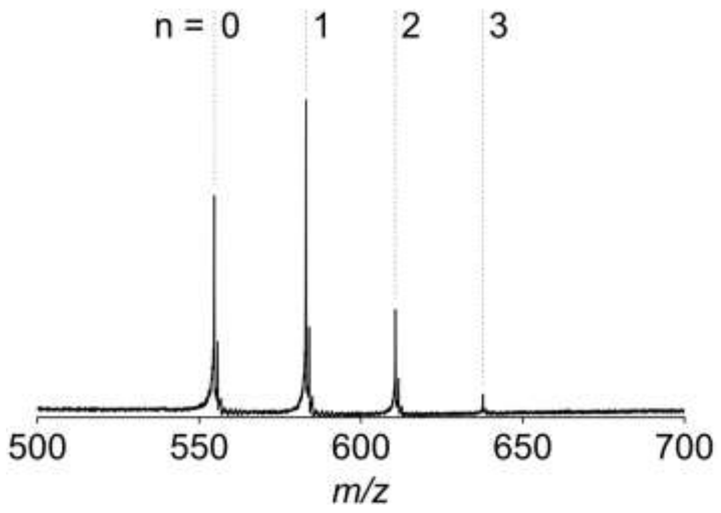

Figure S5. Tagging mass spectra acquired on deprotonated leucine enkephalin ions in negative ion mode. The numbers above each peak denote the number of N_2 tags on each ion.
